# In vitro effects of hyaluronic acid on human periodontal ligament cells

**DOI:** 10.1186/s12903-017-0341-1

**Published:** 2017-01-16

**Authors:** Masako Fujioka-Kobayashi, Heinz-Dieter Müller, Andrea Mueller, Adrian Lussi, Anton Sculean, Patrick R. Schmidlin, Richard J. Miron

**Affiliations:** 1 Department of Cranio-Maxillofacial Surgery, Bern University Hospital, Inselspital, Bern, Switzerland; 2 Department of Oral Surgery, Institute of Biomedical Sciences, Tokushima University, Tokushima, Japan; 3 Department of Preventive, Restorative and Pediatric Dentistry, School of Dental Medicine, University of Bern, Bern, Switzerland; 4 Clinic of Preventive Dentistry, Periodontology and Cariology, Center of Dental Medicine, University of Zurich, Zürich, Switzerland; 5 Department of Periodontology, School of Dental Medicine, University of Bern, Bern, Switzerland; 6 Department of Periodontology, College of Dental Medicine, Nova Southeastern University, Fort Lauderdale, FL USA; 7 Cell Therapy Institute, Center for Collaborative Research, Nova Southeastern University, Fort Lauderdale, FL USA; 8 Department of Periodontics and Oral Medicine, University of Michigan School of Dentistry, Ann Arbor, MI USA

**Keywords:** Hyaluronic acid, Hyaluronan, Periodontal regeneration, Soft tissue regeneration, Connective tissue regeneration

## Abstract

**Background:**

Hyaluronic acid (HA) has been reported to have a positive effect on periodontal wound healing following nonsurgical and surgical therapy. However, to date, a few basic *in vitro* studies have been reported to investigating the potential of HA on human periodontal ligament (PDL) cell regeneration. Therefore, the aim of this study was to investigate the effect of HA on PDL cell compatibility, proliferation, and differentiation in vitro.

**Methods:**

Either non-cross-linked (HA_ncl) or cross-linked (HA_cl) HA was investigated. Human PDL cells were seeded in 7 conditions as follows (1) Control tissue culture plastic (TCP) (2) dilution of HA_ncl (1:100), (3) dilution of HA_ncl (1:10), 4) HA_ncl directly coated onto TCP, (5) dilution of HA_cl (1:100), 6) dilution of HA_cl (1:10) and (7) HA_cl directly coated onto TCP. Samples were then investigated for cell viability using a live/dead assay, an inflammatory reaction using real-time PCR and ELISA for MMP2, IL-1 and cell proliferation via an MTS assay. Furthermore, the osteogenic potential of PDL cells was assessed by alkaline phosphatase(ALP) activity, collagen1(COL1) and osteocalcin(OCN) immunostaining, alizarin red staining, and real-time PCR for genes encoding Runx2, COL1, ALP, and OCN.

**Results:**

Both HA_ncl and HA_cl showed high PDL cell viability (greater than 90%) irrespective of the culturing conditions. Furthermore, no significant difference in both mRNA and protein levels of proinflammatory cytokines, including MMP2 and IL-1 expression was observed. Both diluted HA_ncl and HA_cl significantly increased cell numbers compared to the controlled TCP samples at 3 and 5 days. HA_ncl and HA_cl in standard cell growth media significantly decreased ALP staining, COL1 immunostaining and down-regulated early osteogenic differentiation, including Runx2, COL1, and OCN mRNA levels when compared to control samples. When osteogenic differentiation medium (ODM) was added, interestingly, the expression of early osteogenic markers increased by demonstrating higher levels of COL1 and ALP expression; especially in HA 1:10 diluted condition. Late stage osteogenic markers remained inhibited.

**Conclusions:**

Both non-cross-linked and cross-linked HA maintained high PDL cell viability, increased proliferation, and early osteogenic differentiation. However, HA was consistently associated with a significant decrease in late osteogenic differentiation of primary human PDL cells. Future in vitro and animal research is necessary to further characterize the effect of HA on periodontal regeneration.

## Background

Hyaluronic acid (HA; also termed hyaluronan or hyaluronate) is an anionic, nonsulfated glycosaminoglycan and considered an optimal biomaterial for tissue engineering, given its broad expression in a connective tissue as well as the significant role it plays during organogenesis, cell migration and development in general [1–4]. Non-cross-linked HA (HA_ncl) is biodegradable, biocompatible, bioresorbable and also well known to improve tissue lubrication in cartilage, guides cell growth and differentiation, and speeds the healing and repair of chronic wounds [5]. Cross-linked HA (HA_cl) has also been utilized for tissue engineering as a scaffold to further improve the overall mechanical performance of the scaffolding material and rigidity supporting the growth of various cells [6–9]. HA has been widely used for patients with knee osteoarthritis due to its ability to provide cartilage tissue integrity [10, 11]. Furthermore, in the oral maxillofacial area, HA injections have been used as a treatment option to manage symptoms of temporomandibular joint disorders [12, 13]. More recently, HA has also been utilized for applications for aesthetic purposes in the oral facial regions primarily to reduce or to eliminate facial creases, interdental papilla loss, and various other abnormalities [8, 14, 15].

Due to the growing use of HA in dentistry, HA has also been hypothesized to have influences on periodontal regeneration [16]. The management of periodontal defects is mainly a result of the cooperative treatment of three unique tissues which comprises the periodontium, the periodontal ligament and the cementum and alveolar bone. [6]. HA is an essential component of the periodontal ligament matrix and has been shown to play various important roles in cell adhesion, migration and differentiation mediated by various HA binding proteins and cell-surface receptors such as CD44 [17]. This CD44 antigen is expressed in periodontal tissues and HA-CD44 interaction has been associated with periodontal ligament (PDL) cell proliferation and mineralization activities [18].

Furthermore, other advantages of HA include its anti-inflammatory activity promoting soft and hard tissue healing response, which may be of significant interest during periodontal regeneration [19]. Based on these assumptions, exogenous HA has already been tested in patients with chronic periodontitis in several clinical studies reporting the beneficial effects of HA on reducing bleeding of probing scores and probing depths [2, 20–23]. However, to date, the in vitro effect of HA on periodontal ligament activity has not been clearly investigated.

Therefore, the aim of the present study was to investigate the effects of HA_ncl and HA_cl on PDL cells by stimulating cells under 3 different conditions of either diluted samples with HA (co-existing at 2 concentrations of 1:10 and 1:100 dilutions) or directly by precoating HA onto tissue culture plastic. The PDL cells cultured with either HA_ncl or HA_cl were assessed for cell viability at 24 h, inflammatory cytokines expression at 1 day, cell proliferation at 1, 3 and 5 days and osteogenic differentiation marker expression at 7 days and 14 days.

## Methods

### Reagents and cell culture

HA was kindly provided by Regedent (Zürich, Switzerland) utilizing 2 compositions of HA including non-cross-linked native HA (hyaDENT, BioScience GmbH, Dümmer, Germany) as well as a cross-linked HA (hyaDENT BG, BioScience GmbH). HyaDENT (HA_ncl) contains a formulation of 14.0 mg/mL of sodium hyaluronate (synthesized by bacterial fermentation in *Streptococcus* [24], non-cross-linked), and hyaDENT BG (HA_cl) contains 2.0 mg/mL of sodium hyaluronate and 16.0 mg/mL of sodium hyaluronate cross-linked with butanediol diglycidyl ether (BDDE). The seven groups were tested as follows; (1) control tissue culture plastic (TCP) (2) dilution of HA_ncl (1:100), (3) dilution of HA_ncl (1:10), (4) HA_ncl directly coated onto TCP, (5) dilution of HA_cl (1:100), (6) dilution of HA_cl (1:10) and (7) HA_cl directly coated onto TCP based on our previous report [25]. In short, HA was diluted in standard cell culture growth medium consisting of DMEM (Gibco), 10% fetal Bovine serum (FBS; Gibco) and 1% antibiotics (Gibco). The 100 μl of HA were directly pre-coated in per 24-culture well and then the amount of HA was adjusted the same between 1:10 dilution and coating conditions per well in the end post cell seeding.

The primary human PDL cells were obtained from the middle third portion of each tooth extracted from three healthy patients, with no signs of periodontal disease extracted for orthodontic as previously described [26, 27]. Using discarded tissue for research purposes was approved by the Ethical commission of the Canton Bern without an IRB. All patients gave their consent. Briefly, PDL tissues isolated from the center of the root surface with a surgical scalpel were minced, transferred to TCP with media changes every 2 or 3 days. The PDL cells were detached from TCP using 0.25% EDTA-Trypsin (Gibco, Life Technologies, Carlsbad, CA, USA) prior to reaching confluency. Cells used for experimental seeding were from passages 4–6. Cells were cultured in a humidified atmosphere at 37 °C in the cell growth medium. For in vitro experiments, cells were seeded with HA contained within cell culture media at a density of 10,000 cells in 24 well culture plates for cell proliferation experiments and 50,000 cells per well in 24 well dishes for real-time PCR, ELISA, ALP assay, immunostaining and alizarin red experiments. For experiments lasting longer than 5 days, the medium was replaced twice weekly.

### Cell viability

Primary human PDL cells were seeded in at a density of 12,500 cells / cm^2^ with (1) control TCP (2) dilution of HA_ncl (1:100), (3) dilution of HA_ncl (1:10), (4) HA_ncl directly coated, (5) dilution of HA_cl (1:100), (6) dilution of HA_cl (1:10) and (7) HA_cl directly coated, on chamber slides (Sigma, St. Louis, MO, USA). At 1 day post cell seeding, cells were evaluated using a live-dead staining assay according to the manufacturer’s protocol (Enzo Life Sciences AG; Lausen, Switzerland) as previously described [28]. Fluorescent images were quantified with a fluorescent microscope (OLYMPUS BX51, Tokyo, Japan).

### Proliferation assay

PDL cells were seeded in 24-well plates at a density of 10,000 cells per well in a 24 well culture plate with the same conditions, (1) control TCP (2) dilution of HA_ncl (1:100), (3) dilution of HA_ncl (1:10), (4) HA_ncl directly coated onto TCP, (5) dilution of HA_cl (1:100), (6) dilution of HA_cl (1:10) and (7) HA_cl directly coated onto TCP. Cells were quantified using a fluorescent MTS assay (Promega, Madison, WI, USA) at 1, 3 and 5 days for cell proliferation as previously described [29]. At desired time points, cells were washed with phosphate-buffered saline (PBS, pH = 7.4) and quantified using a ELx808 Absorbance Reader (BIO-TEK, Winooski, VT, USA).

### Real-time PCR analysis

PDL cells were first cultured for 1 day with HA in order to investigate its inflammatory marker expressions, including matrix metalloproteinase-2 (MMP2) and interleukin-1 (IL-1). Moreover, in order to investigate the effects of HA on the osteogenic differentiation, the cells were stimulated for 7 days within each concentration of HA with and without osteogenic differentiation medium (ODM), which consisted of DMEM supplemented with 10% FBS, 1% antibiotics, 50 μg/mL ascorbic acid (Sigma) and 10 mM β-glycerophosphate (Sigma) to promote osteogenic differentiation as previously described [30]. The RNA expressions of runt-related transcription factor 2 (Runx2), collagen1a2 (COL1a2), alkaline phosphatase (ALP), osteocalcin (OCN) in either condition were measured. The total RNA was harvested using High Pure RNA Isolation Kit (Roche, Basel, Switzerland). Primer and probe sequences were fabricated with primer sequences according to Table 1. A Nanodrop 2000c (Thermo, Wilmington, DE, USA) was used to quantify the total RNA levels. Real-time RT-PCR was performed using FastStart Universal SYBR Green Master mix (Roche) and quantified on an Applied Biosystems 7500 Real-Time PCR machine. The amplification profile was 40 cycles at 95 °C for 15 s (annealing), followed by 60 °C for 60 s (elongation). The ∆∆Ct method was used to calculate gene expression levels normalized to the expression of glyceraldehyde 3-phosphate dehydrogenase (GAPDH).

**Table 1:** PCR primers for genes encoding MMP2, IL-1, Runx2, ALP, COL1a2, OCN and GAPDH

Gene	Primer Sequence
hMMP2 F	ccccaaaacggacaaaga
hMMP2 R	cttcagcacaaacaggttgc
hIL-1 F	ggttgagtttaagccaatcca
hIL-1 R	ggtgatgacctaggcttgatg
hRunx2 F	tcttagaacaaattctgcccttt
hRunx2 R	tgctttggtcttgaaatcaca
hCOL1a2 F	cccagccaagaactggtatagg
hCOL1a2 R	ggctgccagcattgatagtttc
hALP F	gacctcctcggaagacactc
hALP R	tgaagggcttcttgtctgtg
hOCN F	agcaaaggtgcagcctttgt
hOCN R	gcgcctgggtctcttcact
hGAPDH F	agccacatcgctcagacac
hGAPDH R	gcccaatacgaccaaatcc

### Inflammatory cytokine quantification with ELISA

The supernatant culture media were collected at 1 and 3 days post cell seeding. MMP2 (DY902, range = 0.625–20.00 ng/mL) and IL-1β/IL-F2 (DY201, range = 3.91–250 pg/mL) were quantified using an ELISA assays (R&D Systems, Minneapolis, MN, USA) according to manufacturer’s protocol as previously described [31, 32]. Briefly, 100 μL of assay diluents and 100 μL of sample were incubated for 2 h at room temperature in antibody-precoated 96-well plates. Wells were washed 3 times with washing buffer, incubated for 2 h with peroxidase-conjugated antibody solution, washed again, followed by the addition of 100 μL of substrate solution for 20 min and 50 μL of stopping solution for 20 min. Absorbance was measured at 450 nm and 570 nm on an ELx808 Absorbance Reader and subtract at 570 nm from the readings at 450 nm.

#### ALP activity assay

PDL cells were stimulated within each concentration of HA with and without ODM. At 7 days, cells were quantified for alkaline phosphatase expression utilizing a cell imaging system. Alkaline phosphatase activity was monitored using a Leukocyte alkaline phosphatase kit (procedure No. 86, Sigma) as previously described [33]. PDL cells were fixed by immersion in a citrate-acetone-formaldehyde fixative solution for 5 min. The alkaline-dye mixture was prepared by mixing 1 mL Sodium Nitrite Solution and 1 mL of a fast red violet alkaline solution dissolved in 45 mL of distilled water and 1 mL of Naphtol AS-Bl alkaline solution. Surfaces were then placed in an alkaline dye mixture solution for 15 min protected from light followed by rinsing in deionized water. All images were captured on a Wild Heerbrugg M400 ZOOM Makroskop (WILD HEERBRUGG, Heerbrugg, Switzerland) at the same magnification and light intensity and imported into Image J software (NIH, Bethesda, MD, USA). Image segmentation (Thresholding) was used to generate percent stained values for each field of view.

#### Immunofluorescent staining

At 14 days post PDL cell seeding, the cells were fixed with 4% formaldehyde for 10 min, followed by permeabilized within PBS containing 0.2% Triton X-100 and blocked in PBS containing 1% bovine serum albumin (BSA, Sigma) for 1 h. Subsequently, cells incubated overnight at 4 °C either with a polyclonal rabbit to collagen type I antibody (sc-28657, Santa Cruz, CA, USA) or a polyclonal rabbit to osteocalcin antibody (sc-30044, Santa Cruz) at a dilution of 1:75 in PBS containing 1% BSA. After washing with PBS, cells were incubated for 1 h at 37 °C with TR-conjugated-goat-anti-rabbit antibodies (sc-2780, Santa Cruz) (1:100) diluted in PBS containing 1% BSA. Prior to viewing, samples were mounted with Vectashield containing DAPI nuclear staining (Vector, Burlingame, CA, USA). Images were captured from each surface with an OLYMPUS BX51 fluorescence microscope. The optical density (OD) of the fluorescent staining was quantified from 3 independent experiments using Image J software.

#### Mineralization assay

Alizarin red staining was performed to determine the presence of extracellular matrix mineralization. PDL cells were stimulated for 14 days within each concentration of HA in ODM. After 14 days, cells were fixed in 96% ethanol for 15 min and stained with 0.2% alizarin red solution (Alizarin Red S, Sigma) in water (pH 6.4) at room temperature for 1 h as previously described [30]. All images were captured and the percentage of staining was evaluated in the same manner as the ALP assay.

#### Statistical analysis

All experiments were performed in triplicate with three independent experiments for each condition. Mean and standard error (SE) were analyzed for statistical significance using a one-way analysis of variance with Tukey post hoc test (*, *p* values < 0.05 was considered significant) by GraphPad Prism 6.0 software (GraphPad Software, Inc., La Jolla, CA, USA).

## Results

### Cell viability and proliferation in response to HA

**Fig. 1:**
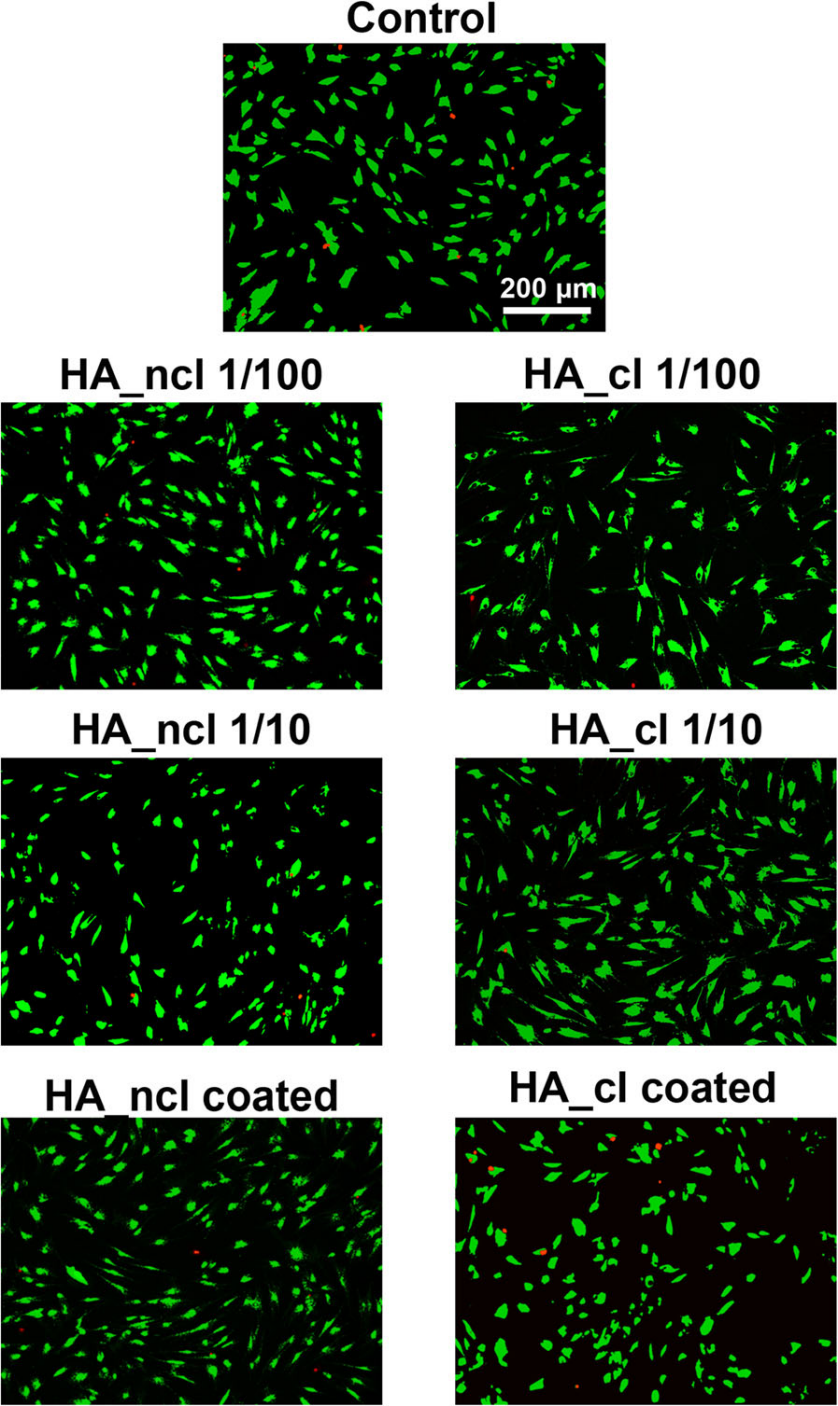
Cell viability staining of primary human primary PDL cells exposed to control (TCP), non-cross-linked HA (HA_ncl) and cross-linked HA (HA_cl) surfaces. For cell viability, Live-Dead staining was done with viable cell appearing in green and dead cells in red. The results from these experiments demonstrated that both HA_ncl and HA_cl are highly biocompatible at dilutions of 1:100 and 1:10 as well as pre-coated onto cell culture plastic

**Fig. 2:**
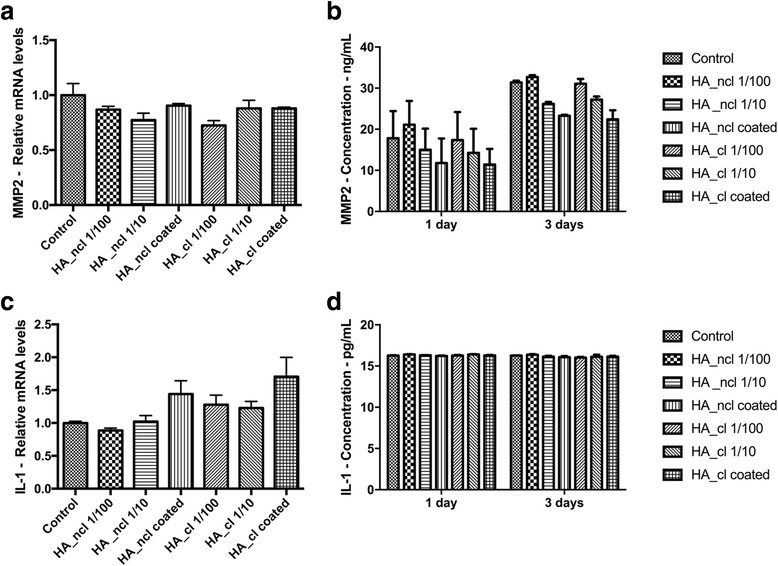
Real-time PCR of PDL cells seeded with HA_ncl and HA_cl for genes encoding (**a**) matrix metalloproteinase-2 (MMP2), (**c**) Interleukin-1 (IL-1), at 1 days post seeding. Protein release at 1 and 3 days of (**b**) MMP2, (**d**) IL-1, (** denotes significantly higher than all other modalities among HA treated groups, *p* < 0.05)

**Fig. 3:**
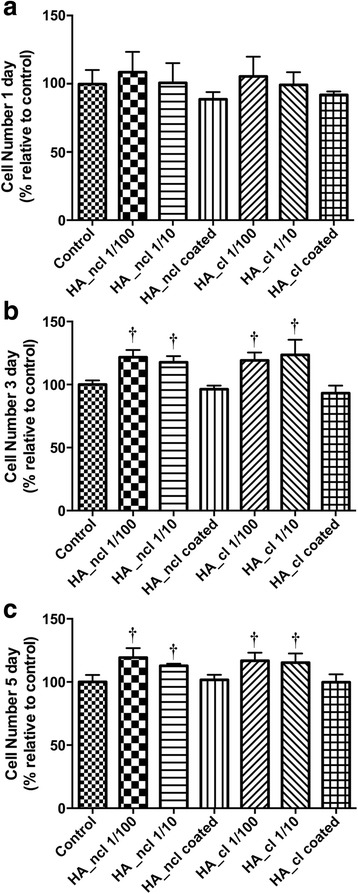
Proliferation assay of PDL cells seeded with HA_ncl and HA_cl at (**a**) 1, (**b**) 3 and (**c**) 5 days post seeding. It was found that both HA and HA_cl at dilutions of 1:100 and 1:10 significantly increased cell numbers at 3 days and 5 days post seeding when compared to control samples († denotes significantly higher than control, *p* < 0.05)

To investigate the biocompatibility of HA towards human PDL cells, the alive / dead assay was utilized. It was determined that both HA_ncl or HA_cl maintained a high level of cell viability (over 90%) in the presence of HA at various concentrations (Fig. 1). Moreover, the mRNA expression of inflammatory cytokine genes encoding MMP2 and IL-1 of PDL cells treated with either HA_ncl or HA_cl demonstrated little differences at 1 day post seeding when compared to control samples (Fig. 2a, c). Inflammatory cytokine release of MMP and IL-1 from PDL cells treated with either HA_ncl or HA_cl was investigated at 1 day and 3 days post cell seeding (Fig. 2b, d). There was no significant difference between MMP2 and IL-1 release among both HA_ncl and HA_cl treated groups as well as mRNA expression (Fig. 2b, d). Thereafter, PDL cell proliferation was investigated in response to HA_ncl and HA_cl at 1, 3 and 5 days post seeding (Fig. 3). It was found that while cell numbers were indifferent at 1 day post seeding (Fig. 3a), a significant increase was observed at 3 and 5 days in either diluted condition for both HA_ncl and HA_cl (Fig. 3b, c).

### Cell differentiation in response to HA

**Fig. 4:**
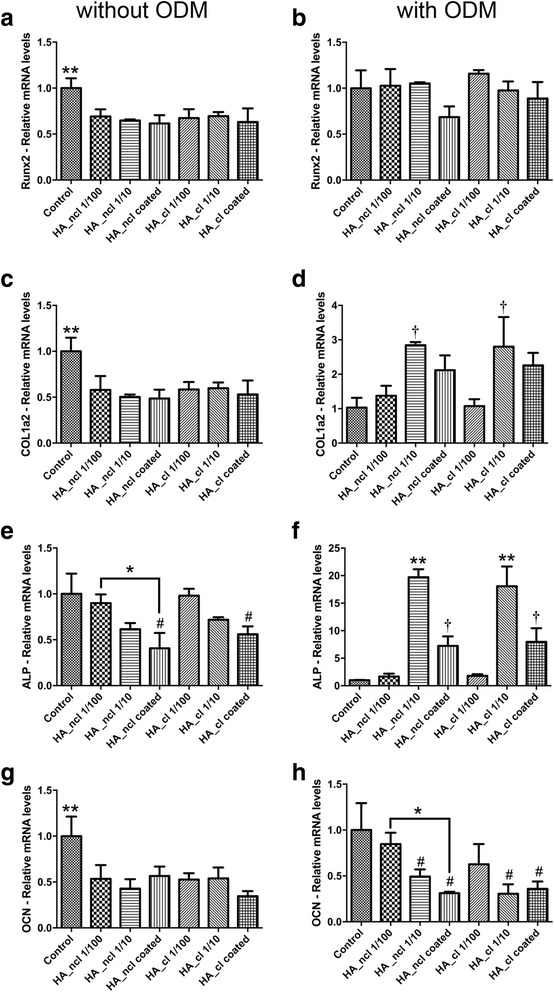
Real-time PCR of PDL cells seeded with HA_ncl and HA_cl treatment for genes encoding (**a**, **b**) Runx2, (**c**, **d**) Collagen 1 alpha 2 (COL1a2), (**e**, **f**) alkaline phosphatase (ALP) and (**g**, **h**) osteocalcin (OCN) at 7 days post seeding. Cells were treated (**a**, **c**, **e**, **g**) in regular growth medium or (**b**, **d**, **f**, **h**) with ODM. (* denotes significant difference, *p* < 0.05; # denotes significantly lower than control, *p* < 0.05; † denotes significantly higher than control, *p* < 0.05; ** denotes significantly higher than all other treatment modalities, *p* < 0.05)

**Fig. 5:**
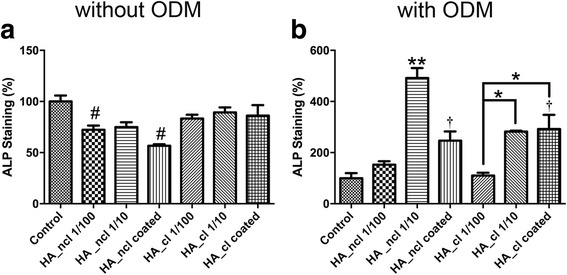
Alkaline phosphatase staining of PDL cells treated by HA_ncl and HA_cl (**a**) in growth medium or (**b**) within ODM at 7 days post seeding. Both HA_ncl and HA_cl significantly decreased ALP staining without ODM while with ODM both HA_ncl and HA_cl significantly increased ALP staining when compared to control samples (* denotes significant difference, *p* < 0.05; # denotes significantly lower than control, *p* < 0.05; † denotes significantly higher than control, *p* < 0.05; ** denotes significantly higher than all other treatment modalities, *p* < 0.05)

**Fig. 6:**
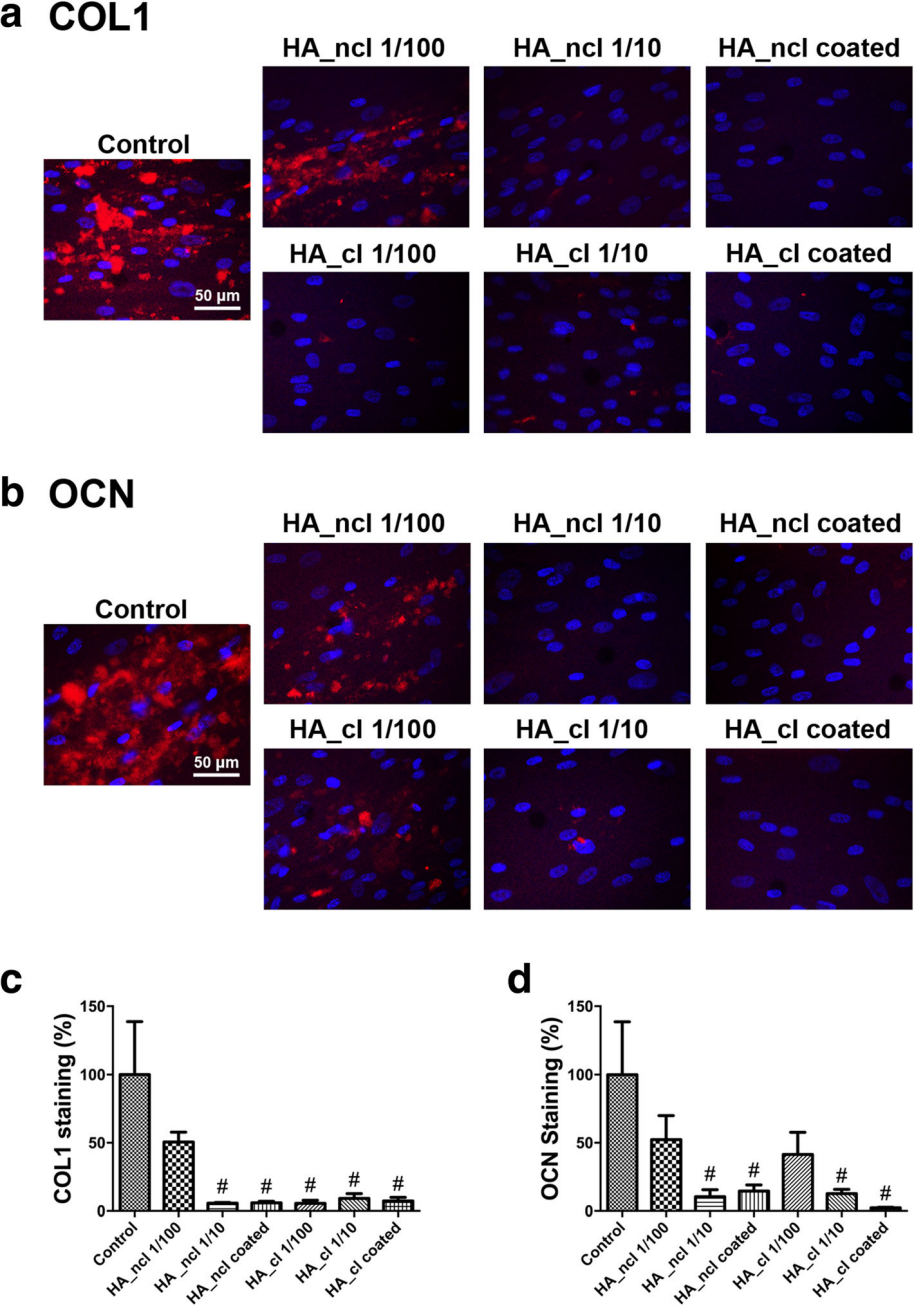
Immunofluorescent COL1 staining and OCN staining at 14 days post cell seeding with HA_ncl and HA_cl. (**a**) The merged images of immunofluorescent detection of COL1 (red) and DAPI (blue). (**b**) The merged images of immunofluorescent detection of OCN (red) and DAPI (blue). (**c**, **d**) Quantified data of (**c**) COL1 and (**d**) OCN immunostaining at 14 days (# denotes significantly lower than control, *p* < 0.05)

**Fig. 7:**
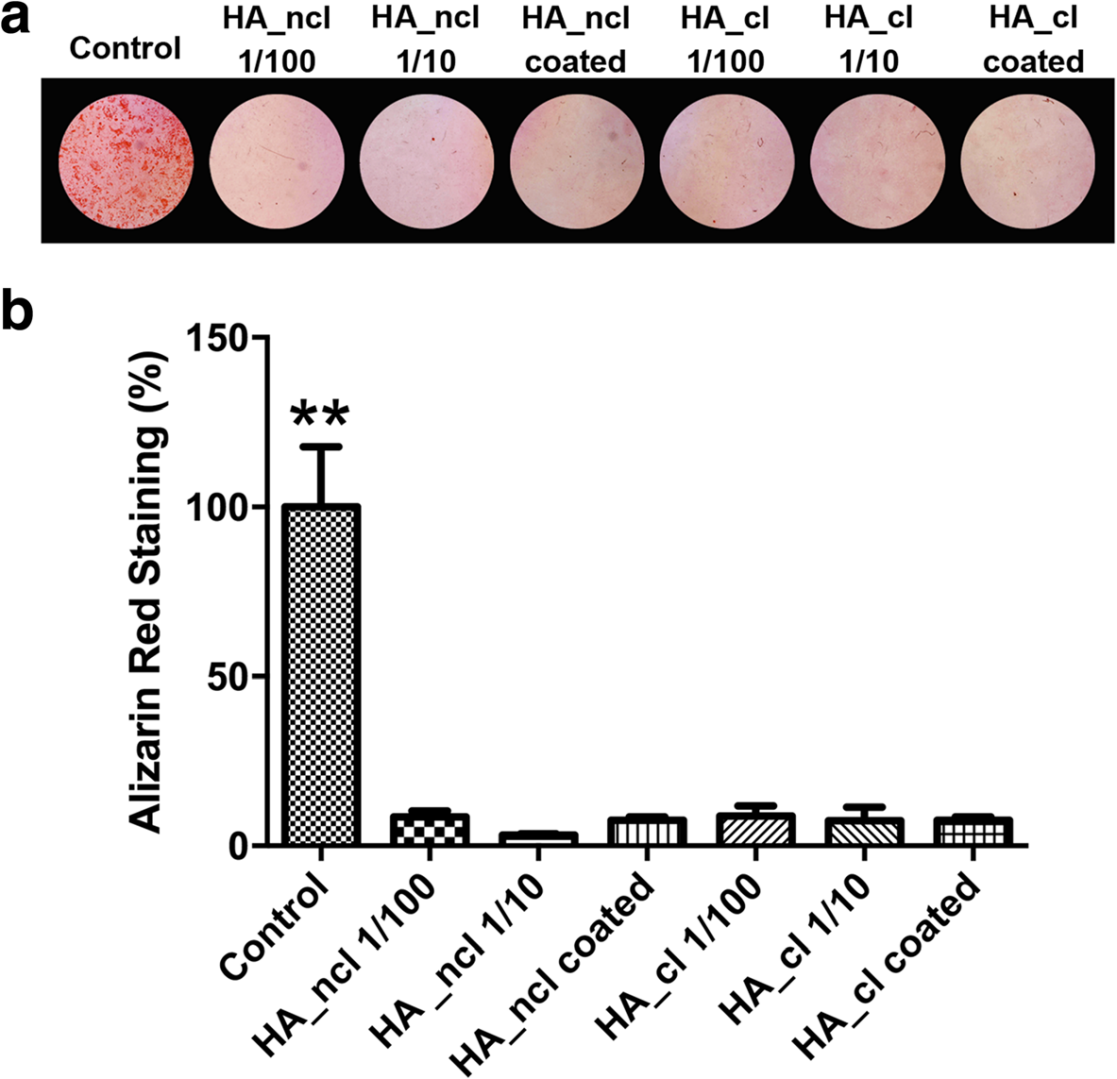
Alizarin red staining denoting mineralization at 14 days post seeding. (**a**) Alizarin red staining images and (**b**) quantified data of alizarin red staining from colour thresholding software for PDL cells treated with HA_ncl or HA_cl (** denotes significantly higher than all other treatment modalities, *p* < 0.05). It was found that both HA_ncl and HA_cl treatment significantly decreased alizarin red staining

Thereafter, the primary human PDL cells were investigated for their ability to differentiate when cultured with HA_ncl and HA_cl (Figs. 4, 5, 6 and 7). The mRNA expression of osteogenic markers was compared by real-time PCR for genes encoding Runx2, COL1a2, ALP and OCN at 7 days (Fig. 4). All HA_ncl and HA_cl treatments significantly downregulated Runx2, COL1a2, ALP and OCN mRNA levels when compared with control samples (Fig. 4a, c, e, g), in normal growth medium. When ODM supplement was added to culture media, it was found that no significant difference was observed in the Runx2 mRNA expression (Fig. 4b), whereas both HA_ncl and HA_cl in 1:10 diluted condition demonstrated a 3-fold significant increase in COL1a2 mRNA levels (Fig. 4d) and a 20-fold increase in ALP levels. Nevertheless, the OCN mRNA expression was still significantly downregulated in either 1:10 diluted or coated conditions for both HA_ncl and HA_cl despite culture with ODM at 7 days post cell seeding (Fig. 4h).

Moreover, it was generally found that HA_ncl significantly decreased ALP staining. HA_cl treatment demonstrated no significant changes in the ALP staining at 7 days likely as a result of the cross-linking HA (Fig. 5a). Interestingly, when ODM was added to cell culture media to promote osteogenic differentiation, HA_ncl at a dilution of 1:10 significantly increased up to 5-fold ALP staining when compared to all other treatment modalities (Fig. 5b, depicted by **). In addition, both HA_ncl and HA_cl coated samples demonstrated a significant increase in ALP staining under ODM supplement when compared to control TCP at 7 days (Fig. 5b). Thereafter, it was demonstrated that both HA_ncl and HA-cl significantly decreased COL1 immunofluorescent staining at 14 days post seeding (Fig. 6a, c) as well as mRNA expression via real-time PCR at 7 days (Fig. 4c).

Cells were further investigated by OCN immunostaining and alizarin red staining when induced by HA_ncl and HA_cl treatment (Figs. 6b, d, and 7). All HA_ncl and HA_cl treatment significantly decreased OCN protein expression and alizarin red staining when compared to control samples as well as OCN mRNA expression (Fig. 4g, h).

## Discussion

In the present study, the effect of high molecular weight (HMW) HA_ncl and HA_cl were evaluated on in vitro periodontal ligament cell behavior. It was first found that both HA_ncl and HA_cl did not elucidate PDL cell apoptosis even at high concentrations (Fig. 1). Both compositions of HA were shown to be biocompatible on PDL cells without any noticeable differences between their in vitro conditions (diluted in culture media versus pre-coated onto TCP).

We then sought to investigate the inflammatory response of HA on PDL cells. It has previously been shown that low molecular weight (LMW, 100–500 kDa) HA, but not the native HMW HA molecules (~4,000 kDa), stimulated inflammatory cells [34]. Nakatani et al. reported that the expression of MMP-1 in cultured human PDL cells was enhanced by the treatment with HA-oligo through p38MAPK signaling pathway, suggesting that the degradation of the periodontal tissues under pathologic conditions may involve MMP-1 induction by HA-oligo [35]. On the other hand, the majority of products used in connection with periodontal therapy contain HMW HA [8]. The mechanism of anti-inflammatory effects of HMW HA has been widely investigated previously due to the pronounced impact and desire for treatment modalities in the field of osteoarthritis and periodontitis. HA has been shown to modulate inflammation in articular chondrocytes and synoviocytes, due to the specific inhibition of MMPs [19, 36] and down-regulation of TNF-α, IL-8, and inducible nitric oxide synthase [37]. In the present study, HMW HA and HA_cl did not affect the mRNA expressions of inflammatory cytokines, including MMP-2 and IL-1 at 1 day post seeding in healthy PDL cells (Fig. 2). The present study was assessed only in utilizing healthy human PDL cells, whereas in the presence of inflammation, HMW HA within these tissues is reportedly broken down to LMW HA by reactive oxygen species or by bacterial hyaluronidase [8, 38–40]. Therefore, these combined results suggest that although HA demonstrated the little influence on the inflammation in the current study utilizing healthy PDL cells, a more pronounced effect could be observed in diseased tissues due to gingival tissue inflammation in periodontitis.

Thereafter, the effects of HA demonstrated an increase in PDL cell proliferation (Fig. 3). During the granulation phase of periodontal tissue repair, HA has been shown to be a key protein highly expressed in various tissues responsible for promoting cell proliferation, migration, and granulation tissue organization [19]. In non-mineralized tissues, HA is transiently elevated during the formation of tissue repair and helps with the re-establishment of the epithelium [38]. In our study, both 1:100 and 1:10 dilution of HA_ncl, as well as HA_cl, promoted cell proliferation at 3 and 5 days post PDL cell seeding (Fig. 3). It was previously shown by Takeda et al. in the same manner that HMW HA enhanced cell adhesion and proliferation of human periodontal ligament cells [9]. In our study, both diluted HA compositions and pre-coated HA promoted PDL cell proliferation, which suggests a positive effect for periodontal tissue regeneration.

The effect of HMW HA on cell differentiation has however had controversial findings. To date, most studies reported that cell differentiation was increased by low-MW HA [16, 41–43]. Huang et al. [41] reported an osteogenic cell behavior of HMW HA in a concentration-dependent manner on rat mesenchymal stem cells. Moreover, it was demonstrated that sulfated HMW HA could enhance the osteogenic differentiation of human mesenchymal stem cells [44]. On the other hand, studies have also shown that cell differentiation was not affected by HMW HA [16, 45–48]. For example, Kaneko et al. demonstrated that HA inhibited BMP-induced osteoblastic differentiation through the CD44 receptor in osteoblasts [45]. Noteworthy, in a pure bone defect model, it was demonstrated that HA-gelatin hydrogels loaded into biphasic calcium phosphate (BCP) ceramics in rabbit femurs promoted the new bone formation and collagen mineralization [46]. HA gel has further been shown to accelerate the healing process in the tooth sockets of rats, stimulating the expression of osteogenic proteins such as bone morphogenetic protein (BMP)-2 and osteopontin (OPN) in vivo [47]. On the other hand, Atilgan et al. reported that the demineralized bone matrix (DBM) + tricalcium phosphate + HA combination showed more new bone formation without HA [48]. Therefore, there remains great interest to determine what may be causing the reported variability in the literature.

In the present study, the expression of osteogenic markers stimulated by HA_ncl and HA_cl was investigated with and without ODM. Both HA_ncl and HA_cl down-regulated Runx2, COL1a2 and OCN mRNA levels, suggesting that HA treatment of PDL cells inhibits osteogenesis in a regular cell culture medium (Fig. 4). Moreover, it was observed that HA significantly decreased ALP activity at 7 days, COL1 and OCN immunofluorescent staining at 14 days when compared to control samples (Figs. 5a, 6). Interestingly, however, when ODM was added to promote osteoblast differentiation, it was found that both HA_ncl or HA_cl in 1:10 diluted condition demonstrated a 3-fold and 20-fold increase in COL1a2 and ALP mRNA levels respectively in ODM (Fig. 4d, f). Moreover, HA_ncl at a dilution of 1:10 demonstrated a 5-fold significant increase in ALP staining compared to control samples in the presence of ODM (Fig. 5b). Therefore, it may be concluded that if granted the right culture conditions, the HA may be used to promote early osteogenic differentiation. Interestingly, however, was the finding that irrespective of the addition of ODM, both HA compositions consistently down-regulated late osteoblast differentiation as assessed by OCN mRNA expression as well as alizarin red staining (Fig. 7).

For periodontal regeneration, HMW HA has previously been investigated in a dog intrabony defect model and neither significant periodontal tissue nor bone tissue regeneration was observed [9]. Interestingly, in another canine model, Kim et al. reported that HA improved wound healing and bone formation of hemisection performed extraction sockets with communication of the periodontal lesion [49]. The effect of HMW-HA on periodontal regeneration remains somewhat controversial, however, its contradiction might be explained due to the variety of HA molecular weight, modification methods, concentration, existence of inflammation and also cell types. It may be that HA requires an osteoconductive matrix during periodontal regeneration to improve the osteogenic phase of PDL regeneration, however, this hypothesis certainly requires further investigation.

## Conclusion

Both HA_ncl and HA_cl were shown to be extremely biocompatible and at both concentrations were associated with a significant increase in PDL cell numbers. The early osteogenic differentiation markers such as Runx2, COL1 and ALP were significantly downregulated in standard cell growth media. Interestingly, with the addition of ODM, the expression of early osteogenic markers was shown to significantly increase COL1 and ALP levels, especially at higher concentration (1:10) utilizing both HA_ncl and HA_cl diluted condition. Nevertheless, the late stage osteogenic marker such as OCN as well as calcium formation was inhibited regardless of the ODM addition. Future in vitro models including in inflammatory conditions, as well as animal research, are necessary to further characterize the optimal uses as well as delivery systems of HA for improved clinical use.

## Abbreviations

ALP: Alkaline phosphatase
BCP: Biphasic calcium phosphate
BDDE: Butanediol diglycidyl ether
BSA: Bovine serum albumin
COL: Collagen
DBM: Demineralized bone matrix
DMEM: Dulbecco’s modified eagle medium
EDTA: Ethylenediaminetetraacetic acid
FBS: Fetal Bovine serum
GAPDH: Glyceraldehyde 3-phosphate dehydrogenase
HA: Hyaluronic acid
HA_cl: Cross-linked HA
HA_ncl: Non-cross-linked hyaluronic acid
HMW: High molecular weight
IL-1: Interleukin-1
LMW: Low molecular weight
MMP2: Matrix metalloproteinase-2
MTS: 3-(4,5-dimethylthiazol-2-yl)-5-(3-carboxymethoxyphenyl)-2-(4-sulfophenyl)-2H-tetrazolium
OCN: Osteocalcin
OCN: Osteocalcin
OD: Optical density
ODM: Osteogenic differentiation medium
OPN: Osteopontin
PBS: Phosphate buffered saline
PDL: Periodontal ligament
RT-PCR: Reverse transcription polymerase chain reaction
Runx2: Runt-related transcription factor 2
SE: Standard error
TCP: Tissue culture plastic.
